# Life Cycle, PESTLE and Multi-Criteria Decision Analysis of Membrane Contactor-Based Nitrogen Recovery Process

**DOI:** 10.3390/membranes13010087

**Published:** 2023-01-10

**Authors:** Judit Nagy, Huyen Trang Do Thi, Andras Jozsef Toth

**Affiliations:** Environmental and Process Engineering Research Group, Department of Chemical and Environmental Process Engineering, Budapest University of Technology and Economics, Műegyetem rkp. 3, H-1111 Budapest, Hungary

**Keywords:** life cycle assessment, membrane gas separation, nitrogen recovery, pestle, multi-criteria decision analysis

## Abstract

Nitrogen is one of the most critical nutrients in the biosphere, and it is an essential nutrient for plant growth. Nitrogen exists in the atmosphere vastly as a gaseous form, but only reactive nitrogen is usable for plants. It is a valuable resource and worth recovering in the wastewater sector. The aim of this work was to prepare a comprehensive environmental analysis of a novel membrane contactor-based process, which is capable of highly efficient nitrogen removal from wastewater. Life cycle assessment (LCA), PESTLE and multi-criteria decision analysis (MCDA) were applied to evaluate the process. The EF 3.0 method, preferred by the European Commission, IMPACT World+, ReCiPe 2016 and IPCC 2021 GWP100 methods were used with six different energy resources—electricity high voltage, solar, nuclear, heat and power and wind energy. The functional unit of 1 m^3^ of water product was considered as output and “gate-to-gate” analysis was examined. The results of our study show that renewable energy resources cause a significantly lower environmental load than traditional energy resources. TOPSIS score was used to evaluate the alternatives in the case of MCDA. For the EU region, the most advantageous option was found to be wind energy onshore with a score of 0.76, and the following, nuclear, was 0.70.

## 1. Introduction

To help to achieve the Sustainable Development Goal (SDG) 2—to end hunger and all forms of malnutrition by 2030—nitrogen and phosphorus can increase crop yield in agriculture, even though the uptake of fertilizers by plants is limited and a part of them is lost to the environment [1]. Nitrogen is a crucial for plant growth [2]; it is a renewable gaseous resource, enormously present in the atmosphere [3]. In the atmosphere, only reactive nitrogen is usable for plants [2].

Nitrogen has begun to be considered a valuable resource worth recovering in the wastewater sector. Nitrogen is a critical element in many fertilizers, and it is a crucial nutrient for many agricultural crops [4]. However, transforming the unreactive atmospheric nitrogen to reactive nitrogen during nitrogen fertiliser production is energy-demanding, contributing up to 2% of all energy usage globally [3]. Municipal and industrial wastewater contains many nutrients, for example, nitrogen and phosphorous, as reusable resources [5,6].

In this work, the nitrogen recovery process is in focus. Some technologies used on a large scale are designed for nutrient recoveries, such as membrane technologies, air stripping and struvite precipitation. With the membrane process, the nitrogen can be recovered above 70% and even close to 100% [7]. Vazifehkhoran et al. the (2022) [8] used the air stripping process for NH_3_ recovery from six different slurries. NH_3_ was stripped from the digestate, pig slurry and dairy cattle slurry; for 20 days of the hydraulic retention time (HRT), totals of 92%, 83% and 67% of NH_3_ were stripped, respectively. For 12 days of HRT, total NH_3_ recoveries were 83%, 60% and 41%. The organic N content in most input slurries was in the range of 1.3–1.6 g kg^−1^ [8].

Through phosphate-based recovery processes, such as struvite precipitation, nitrogen can be partly reclaimed from reject waters (<40%) [3]. However, struvite purification is more complex because a large amount of magnesium (Mg^2+^) and phosphate ions (PO_4_^3−^) are needed, resulting in increased costs [9]. Otherwise, both struvite precipitation and ammonia stripping typically require chemical additions, which add to their operational costs [3]. On the other hand, it is not wise to blindly accept all the final end-products achieved in the nitrogen recovery process, because such products may potentially contain a large amount of metal and other contaminants [10].

The wastewater field has transformed from a standard of pollutant removal to resource recovery and the pursuit of circular economies. Therefore, life cycle assessment (LCA) can play an essential role by evaluating the environmental sustainability of new technologies and processes [11]. Over the last 20+ years, there has been serious interest in using LCA in the wastewater field [12]. LCA is a method to investigate and compare different products and services regarding their environmental impacts along the whole life cycle, i.e., from the extraction of raw materials until the end of a product’s life [2]. It is an essential tool for analyzing the environmental impacts of agricultural systems. Lam et al. (2020) [13] recently discussed how LCA methodology has been adapted and applied to evaluating opportunities for wastewater-based nutrient recycling [12].

The gas-permeable membranes have low energy costs compared with traditional processes; therefore, they are considered environmentally benign solutions [14]. The low energy consumption, high selectivity, lower capital cost, simple operation and more efficient gas removal are further advantages of membrane-based gas separation techniques compared with traditional separation operations [15].

## 2. Materials and Methods

### 2.1. Introduction of the Investigated Process

**Figure 1:**
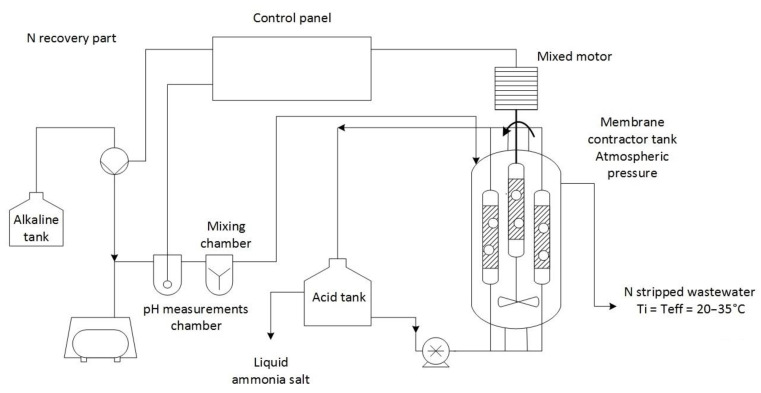
The scheme of the investigated process (amended from [1]).

Figure 1 shows the schematic illustration of the examined N recovery process.

The waste stream was pumped into a pH measurement chamber. The measurement of pH was recorded. A control device sent a command to the alkaline pump to inject enough of the alkaline solution to bring the pH to the desired level for instigating ammonia gas release in the wastewater (pH = 12). After that, the wastewater flowed to the mixing chamber and the membrane contactor tank. The membrane contactor tank contained six membrane modules. Once all the membrane modules were covered with pH-adjusted wastewater, acid circulation and mixing in the membrane contactor tank were initialized [1,16].

The Aeos™ ePTFE hydrophobic, gas-permeable membrane from Zeus Inc. was applied in the investigated process with the following parameters: 10 ± 0.381 mm inside diameter, the internodal distance between 10 and 30 mm, density of 0.45 ± 0.15 g/cm^3^ and 1.0 mm wall thickness [1].

**Figure 2:**
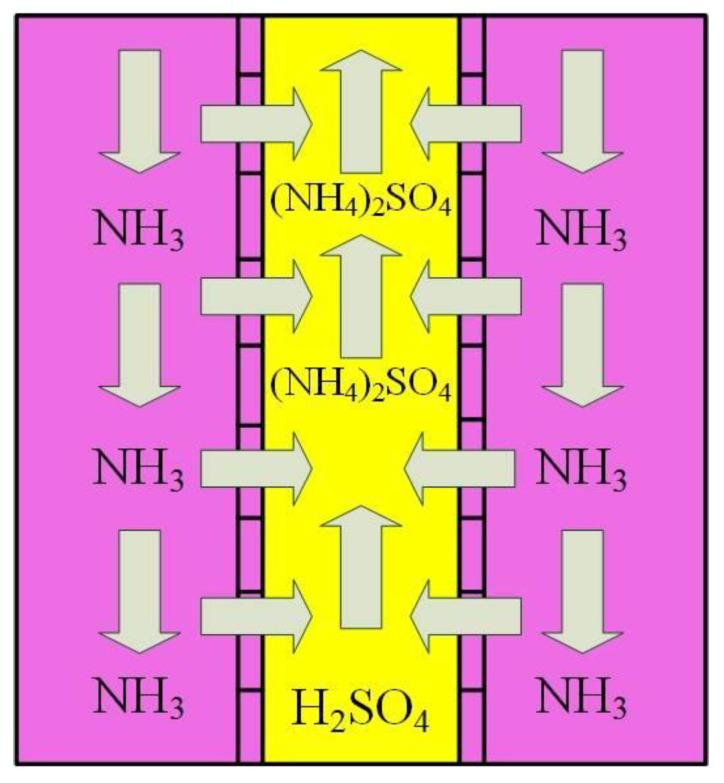
Gas-permeable membrane function principle for ammonia recovery [17].

The ammonia recovery capability of the gas-permeable membrane is based on the following principle. When pH is increased, the ammonium-ammonia balance shifts towards ammonia. Ammonia is a soluble gaseous compound, and it will pass through the membrane. The reason for this because there is always an ammonia concentration gradient over the membrane, depicted in Figure 2. The ammonia inside the membrane reacts with sulphuric acid (H_2_SO_4_) and forms ammonium sulphate ((NH_4_)_2_SO_4_), so the gradient remains constant. In one of our previous works [17], this method has been explained in detail.

The minimum and maximum characteristics in the material of the input waste stream were 822–1250 mg/L total N (mg/L), 9.5–47.3 mg/L total P, 560–4200 mg/L suspended solids (SS), 322–1137 mg/L BOD_7_, 860–2100 mg/L COD_cr_, 7.5–8.0 pH [1]. The main operational parameters of the investigated process were 473 m^3^/year flow rate, 34 kWh/m^3^ energy, 0.7 kg/m^3^ H_2_SO_4_ (98%), 9.2 kg/m^3^ Ca(OH)_2_, 4.6 kg/m^3^ LKD, 5.4 kg/m^3^ and PAX XL 100 (30–40%). The technology achieved a nitrogen removal efficiency of over 90% [1].

María Soto-Herranz et al. (2022) [18] also investigated the gas-permeable tubular membranes for ammonia removal. Ten different configurations were proved to recover ammonia released from pig slurry. From their comprehensive investigation, they determined that an ammonia recovery of up to 91% can be achieved, which confirms the practical application potential of the gas separation membrane process.

### 2.2. Examined Life Cycle Assessment Methods

A life cycle assessment is a systemic tool that assesses the environmental impacts of products and processes across the lifecycle, beginning with raw material acquisition and continuing until their final disposal, which helps determine which product is the least harmful over others [19]. SimaPro software was developed, adding new municipal waste scenarios to all ecoinvent libraries. The municipal waste scenarios present the recycling rates for each type of waste and mix them with the incineration and landfill rates in the corresponding countries. The procedures for each European country are representative of 2019 and are based on Eurostat data. SimaPro version 9.3.0.3 was used as the platform for setting up the model framework for our study. As per the International Organization for Standardization (ISO) 14040 (2006) and ISO 14044 (2006) standards, the LCA model framework consists of four steps: goal and scope definition, life cycle inventory analysis (LCI), life cycle impact assessment (LCIA) and interpretation [20,21].

Life cycle inventory data were converted into a quantitative figure using characterization factors using SimaPro Life Cycle Analysis software version 9.3, registered as a trademark by PRéSustainability B.V. In this study, as a basis for impact analysis and evaluation, the following methods were used: EF 3.0 Method (adapted) V1.02, IMPACT World+Endpoint V1.01, ReCiPe 2016 Endpoint (H) V1.06 and IPCC 2021 GWP 100 (include CO_2_ uptake) V1.00. In the following part, the applied methods for the evaluations are introduced. This study considered the operational phase of the process, which includes the necessary chemical needs and energy demand. This concept is called “gate-to-gate” analysis [22,23]. The base unit of 1 m^3^ of water product was examined as output.

With the Environmental Footprint (EF) database, environmental footprint sector rules can be utilized based on product environmental footprint category rules (PEFCRs) and organization environmental footprint sector rules (OEFSRs). A compatible EF impact assessment method and secondary life cycle inventory datasets are included. Any good or service can be quantified through EF based on its environmental performance throughout its life cycle. The EF 3.0 version is intended to be used in any other PEF/OEF studies outside of the PEFCRs/OEFSRs, as well as to develop new PEFCRs/OEFSRs during the EF transition phase and to implement PEF/OEF studies under any of the PEFCRs/OEFSRs that are being developed. Biogenic CO_2_ uptake and re-emissions are not considered in the Climate Change (biogenic) methodology. Only methane emitted from anaerobic degradation of organic matter in landfills is included in biogenic methane uptake [24,25].

In IMPACT World+, the IMPACT 2002+, LUCAS and EDIP methods are compiled and updated; they have a global scope and are available as midpoints and endpoints (damage level). Most indicators for the different regional impact categories are geographically defined, and each long-term impact category is divided into shorter-term (beyond 100 years after emission) and long-term damage. The suggested version of IMPACT World+ comprises two damage categories: human health and ecosystem quality. Resources and ecosystem services are not considered in the proposed implementation of SimaPro, as the development team regards this as transitional. IMPACT World+ only provides for normalization factors at the damage level, as the development team believes that a science-based mid-point of damage modelling is a more resilient approximation to put into perspective the comparative importance of different mid-point indicators for the same area of protection than any normalization/weighting scheme. The overall global set used to identify the normalization factors is described by a combination of reference years within the 2000 and 2010 periods [24].

The ReCiPe 2016 includes 18 impact categories of midpoints (problem-oriented) and 3 impact categories of endpoints (harm-oriented) from three viewpoints for a global scale: individualism (I), hierarchical (H) and egalitarianism (€). These midpoint impact categories are aggregated into three endpoint categories at the endpoint level: impact on human health, biodiversity and resource scarcity [26].

IPCC 2021 is the follow-up method to IPCC 2013, developed by the Intergovernmental Panel on Climate Change. This method is based on the final governmental dissemination version of the Intergovernmental Panel on Climate Change—IPCC report “AR6 Climate Change 2021: The Physical Science Basis”, which is still subject to duplication, corrections and redistribution. The IPCC 2021 Methods approach has various characterization factors, resulting in six methods for estimating global warming potential (GWP) and two for calculating global temperature potential (GTP). A CO_2_ uptake scenario and a no uptake scenario are included in IPCC 2021 to adapt to varying standards. In addition, three-time horizons are included for GWP [24].

IMPACT Assessment of Chemical Toxics (IMPACT 2002+) is an impact assessment methodology originally developed at the Swiss Federal Institute of Technology—Lausanne (EPFL). The present methodology proposes a feasible implementation of a combined midpoint/damage approach, via 14 midpoint categories to four damage categories. The midpoint categories are the following: human toxicity, respiratory effects, ionizing radiation, ozone layer depletion, photochemical oxidation, aquatic ecotoxicity, terrestrial ecotoxicity, aquatic acidification, aquatic eutrophication, terrestrial acid, land occupation, global warming, non-renewable energy and mineral extraction. The damage categories are human health, ecosystem quality, climate change and resources. The impact assessment method of environmental footprint (EF) was introduced by the European Commission. The EF method is available in two versions, EF 2.0 and EF 3.0. EF 3.0 is the latest version of the method and has numerous changes. EF 3.0 examine the following impact categories: climate change, ozone depletion, human toxicity (cancer), human toxicity (non-cancer), respiratory inorganics, ionising radiation (human health), photochemical ozone formation (human health), acidification, terrestrial eutrophication, freshwater eutrophication, marine eutrophication, land use, ecotoxicity freshwater, water scarcity, resource use (energy carriers) and resource use (mineral and metals). The ReCiPe is the successor of the methods Eco-indicator 99 and CML-IA. ReCiPe implements both strategies and has both midpoint (problem-oriented) and endpoint (damage-oriented) impact categories. At the midpoint level, 18 impact categories are addressed: ozone depletion, human toxicity, ionizing radiation, photochemical oxidant formation, particulate matter formation, terrestrial acidification, climate change, terrestrial ecotoxicity, agricultural land occupation, urban land occupation, natural land transformation, marine ecotoxicity, marine eutrophication, freshwater eutrophication, freshwater ecotoxicity, fossil fuel depletion, minerals depletion and freshwater depletion. The endpoint categories are: human health, ecosystems, resource surplus costs. An update of the method IPCC 2007 was developed by the International Panel on Climate Change. This method lists the climate change factors of IPCC with a timeframe of 20 and 100 years. IPCC characterization factors for the direct (except CH_4_) global warming potential of air emissions. They do not include indirect the formation of dinitrogen monoxide from nitrogen emissions, not accounting for radiative forcing due to emissions of NO_x_, water, sulphate, etc. in the lower stratosphere and upper troposphere [27].

The highest CO_2_ emission is electricity high voltage with the EF 3.0, the IMPACT World+, and the ReCiPe 2016 methods. In comparison, nuclear energy, solar energy, heat and power, and wind energy are 59%, 55%, 50%, and 58% lower than electricity. Based on IPCC 2021 methods, nuclear energy, solar energy, heat and power and wind energy reduce CO_2_ emission by 51%, 47%, 43% and 50% compared with high electricity voltage, respectively. The GWP100-fossils of all energy cases account for the highest proportion, accounting for 70–85% of the total CO_2_ emissions. Overall, heat and power have the lowest CO_2_ emissions and impacts on climate change, followed by solar, wind and nuclear power.

### 2.3. Introduction of the Multi-Criteria Analysis (MCDA)

Multiple criteria must be considered when selecting a wastewater treatment process. The multi-criteria decision analysis (MCDA) method can combine alternative energy sources to evaluate the treatment based on the PESTLE factors: political, economic, social, legal and environmental factors. TOPSIS is a multi-criteria decision-making methodology that takes a fundamental mathematical approximation to optimal alternatives [28,29]. The TOPSIS implementation policy is that the chosen optional object must be the closest to the positive ideal solution and the most distal to the negative ideal solution, incorporating the Euclidean distance for the geometric determination of relative proximity. The positive ideal solution (A+) is the total of the best possible values for each alternative, and the negative ideal solution (A-) contains all the worst possible values for each considered option [30]. These two hypothetical solutions are obtained by calculating the approximation to the positive ideal solution within the process. Based on the relative calculations and comparisons, an alternative priority is chosen [31,32].

The positive and negative ideal solution of the classic TOPSIS process for a single decision maker can be described in a series of the follow-up equations [33]:

(1) $$A^{+}=\left(v_{1}^{+},\;v_{2}^{+},\ldots ,v_{n}^{+}\right)=\{\underset{i}{\max}(w_{j}n_{ij})\}\;$$

(2) $$A^{-}=\underset{i}{\left(v_{1}^{-},\;v_{2}^{-},\ldots ,v_{n}^{-}\right)=\{\underset{i}{\min}\left(w_{j}n_{ij}\right)}\}\;$$

(3) $$v_{ij}=w_{j}n_{ij\;}$$

(4) $$n_{ij}=\frac{x_{ij}}{\sqrt{\sum_{i=1}^{m}x_{ij}^{2}}}$$

where:

$x_{ij}$ is the value of *i*-alternative with respect to *j*-criterion, *i* = 1, 2, … *m*;

$n_{ij}$ is a normalized value, *j* = 1, 2, … *n*

$v_{ij}$ is a weighted normalized value

$w_{j}$ is the weight of the *j*-th criterion, $\overset{n}{\underset{j=1}{\sum }}w_{j}$=1

$(v_{1}^{+}$, $v_{2}^{+},\;v_{n}^{+})\;or\;(v_{1}^{-}$, $v_{2}^{-},\;v_{n}^{-}$) are the maximum or minimum value of the benefit criteria.

The positive ideal solution is calculated of the relative closeness to

(5) $$R_{i}=\frac{\sqrt{\sum_{j=1}^{n}{\left(v_{ij}-v_{j}^{-}\right)}^{2}}\;}{\sqrt{\sum_{j=1}^{n}{\left(v_{ij}-v_{j}^{-}\right)}^{2}}+\sqrt{\sum_{j=1}^{n}{\left(v_{ij}-v_{j}^{+}\right)}^{2}}}$$

where 0 ≤ $R_{i}$ ≤ 1.

Rank the order of preference by choosing the alternative closest to 1.

## 3. Results and Discussion

### 3.1. Demonstrated Results Implemented with LCA Methods

In this research work, EF 3.0 Method (adapted), IMPACT World+ Endpoint (H), ReCiPe 2016 Endpoint (H) and IPCC 2021 methods were investigated. Figure 3, Figure 4, Figure 5, Figure 6, Figure 7, Figure 8, Figure 9, Figure 10, Figure 11 and Figure 12 show the comparison of six different energy sources applied with the previously mentioned methods.

**Figure 3:**
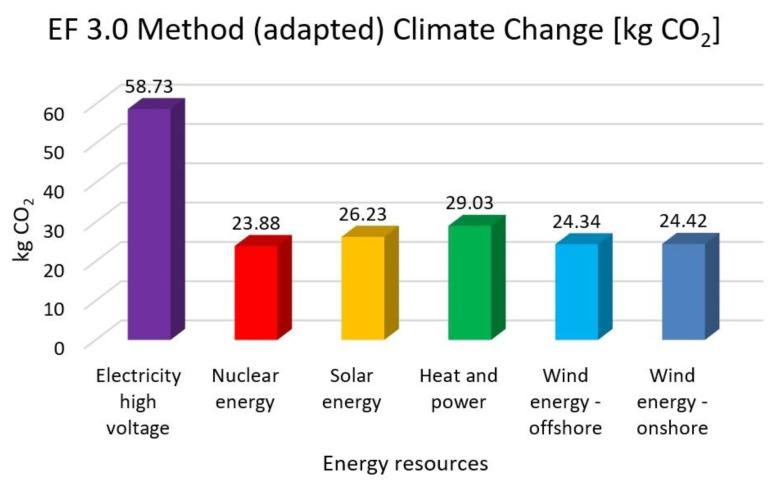
Investigation of climate change with EF 3.0 method (adapted) with different energy sources.

**Figure 4:**
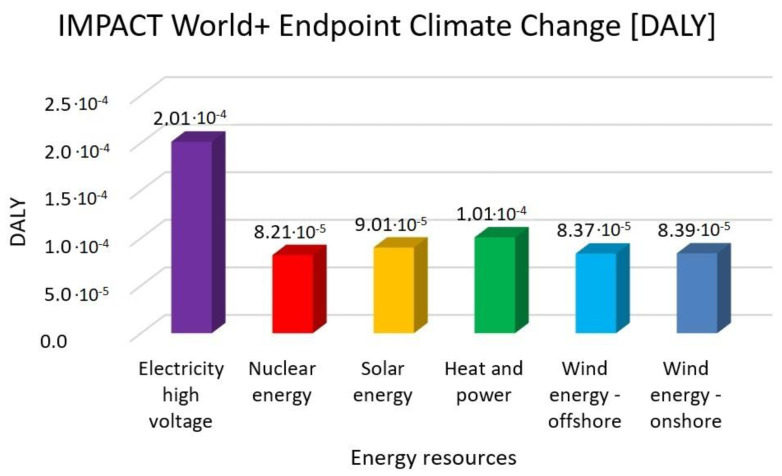
Investigation of climate change with IMPACT World+ endpoint method with different energy sources.

**Figure 5:**
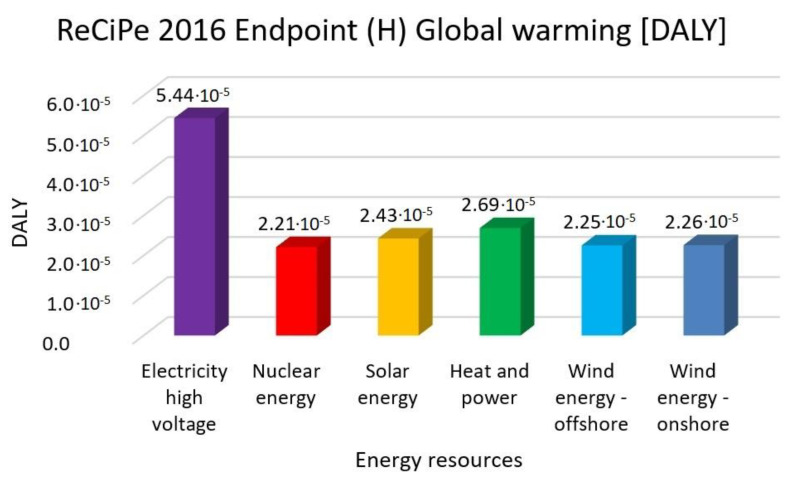
Investigation of global warming with ReCiPe 2016 Endpoint method with different energy sources.

Figure 3, Figure 4 and Figure 5 show the results for climate change and global warming with six different energy sources—electricity high voltage, nuclear energy, solar energy, heat and power, wind energy offshore, and wind energy onshore—investigated with EF 3.0 Method (adapted), IMPACT World+ Endpoint (H) and ReCiPe 2016 Endpoint (H) methods.

As it can be seen in Figure 2, Figure 3 and Figure 4, in climate change, the highest value is electricity high voltage. Nuclear energy is 59% lower, solar energy is 55% lower, heat and power is 50% lower and wind energy is 58% lower than the electricity with the EF 3.0 Method and the IMPACT World+ method. These results are almost two third parts of the electricity high voltage results.

In global warming, the highest value is also electricity high voltage. Nuclear energy is 59%, solar energy is 55%, heat and power is 50% and wind energy is 58% lower than the electricity with the ReCiPe 2016 Method.

**Figure 6:**
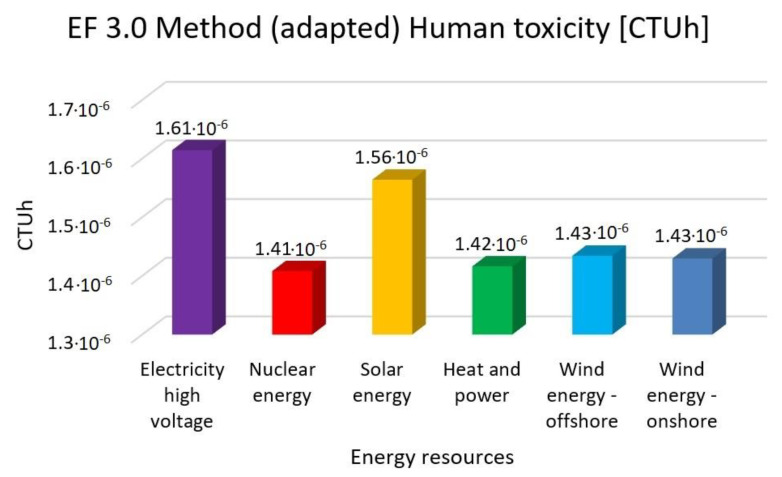
Investigation of human toxicity with EF 3.0 method (adapted) with different energy sources.

**Figure 7:**
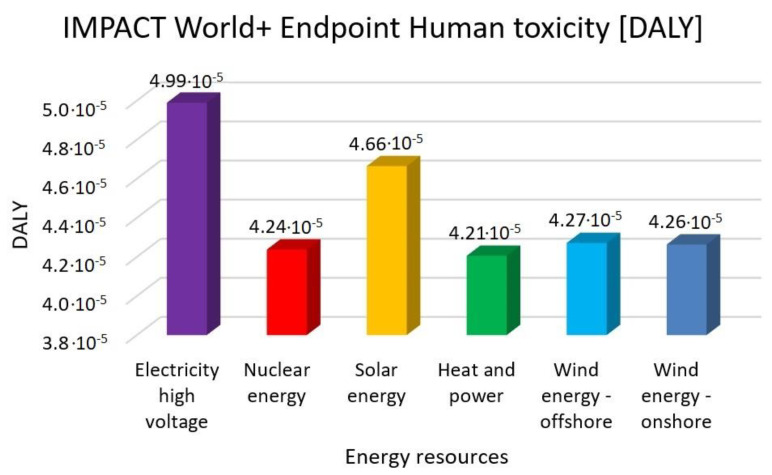
Investigation of human toxicity with IMPACT World+ endpoint method with different energy sources.

**Figure 8:**
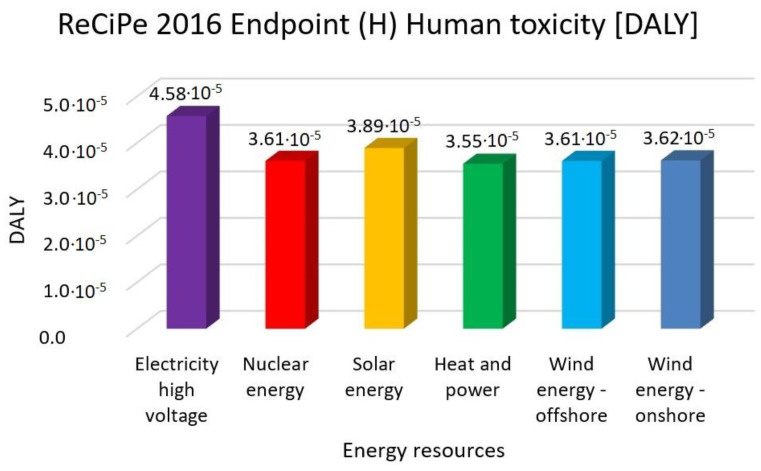
Investigation of human toxicity with ReCiPe 2016 endpoint (H) method with different energy sources.

Figure 6, Figure 7 and Figure 8 show the results of the examination of human toxicity with the same six different energy resources—electricity high voltage, nuclear energy, solar energy, heat and power, wind energy offshore and wind energy onshore—investigated with EF 3.0 Method (adapted), IMPACT World+ Endpoint (H) and ReCiPe 2016 Endpoint (H) methods.

In Figure 5, Figure 6 and Figure 7, the high electricity voltage has the highest value in human toxicity. Nuclear energy is 12%, solar energy is 3%, heat and power is 12% and wind energy is 11% lower than the electricity with the EF 3.0 method. Nuclear energy is 15%, solar energy is 6%, heat and power is 16% and wind energy is 14% lower than the electricity with IMPACT World+ method. Nuclear energy is 21%, solar energy is 15%, heat and power is 22% and wind energy is 21% lower than the electricity with ReCiPe 2016 Method.

**Figure 9:**
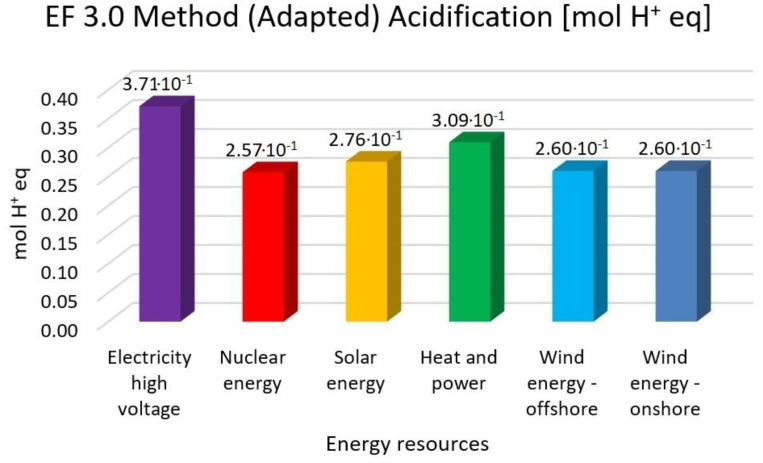
Investigation of acidification with EF 3.0 method (adapted) with different energy sources.

**Figure 10:**
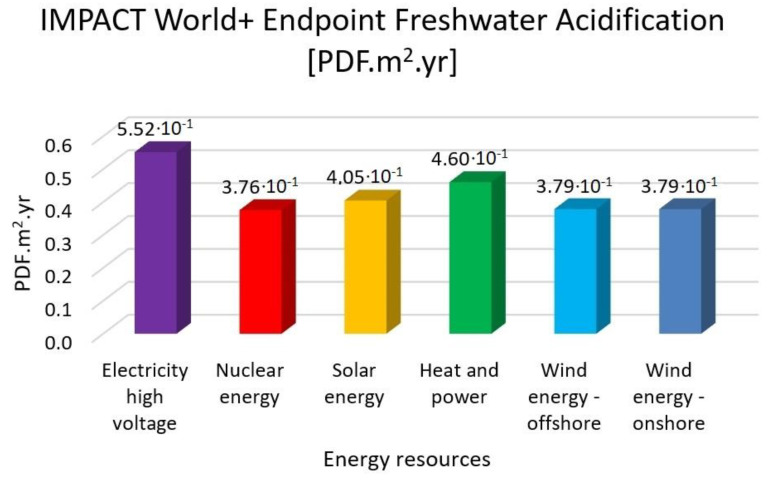
Investigation of freshwater acidification with IMPACT World+ endpoint method with different energy sources.

Figure 8, Figure 9 and Figure 10 show the results of the examination of acidification with high-voltage electricity, nuclear energy, solar energy, heat and power, wind energy offshore and wind energy onshore energy sources, investigated with EF 3.0 Method (adapted), IMPACT World+ Endpoint (H) and ReCiPe 2016 Endpoint (H).

Figure 8, Figure 9 and Figure 10 represent the results for the acidification with the highest value of electricity high voltage. As for the nuclear energy, it is 30% lower; solar energy is 26%, heat and power is 17% and wind energy is 30% lower than the electricity with the EF 3.0 method. Nuclear energy is 37%, solar energy is 40%, heat and power is 46% and wind energy is 38% lower than the electricity with the IMPACT World+ method. Nuclear energy is 30%, solar energy is 25%, heat and power is 16% and wind energy is 30% lower than the electricity with ReCiPe 2016 Method.

**Figure 11:**
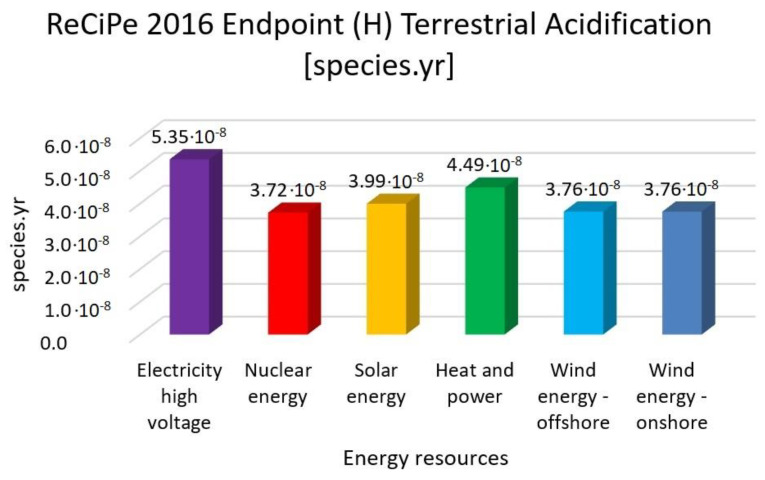
Investigation of terrestrial acidification ReCiPe 2016 endpoint (H) method with different energy sources.

Figure 11 depicts the results for the eutrophication of freshwater with the EF 3.0 method (adapted) with six different energy sources.

It can be realized that nuclear energy is 39%, solar energy is 35%, heat and power is 39% and wind energy is 38% lower than the electricity with EF 3.0 method.

Figure 13, Figure 14, Figure 15, Figure 16 and Figure 17 represent the IPCC 2021 GWP100 Method with six energy resources.

**Figure 12:**
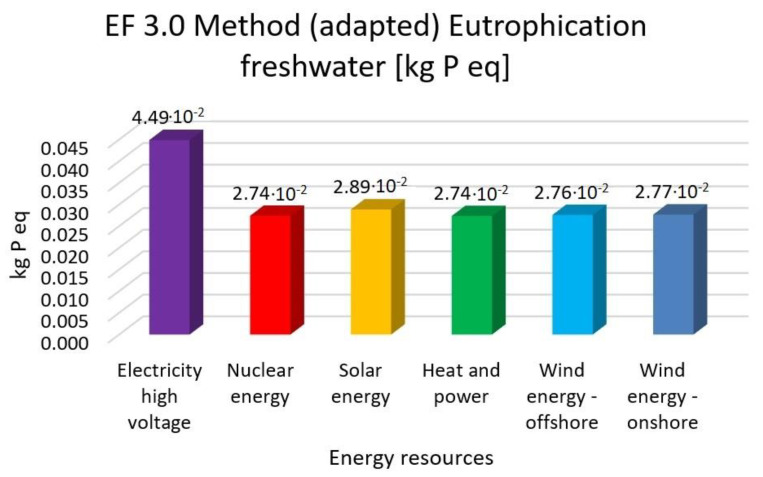
Investigation of eutrophication freshwater with EF 3.0 method with different energy sources.

**Figure 13:**
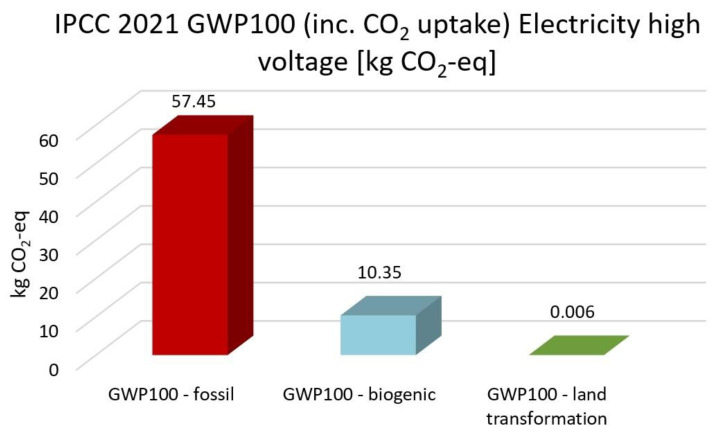
Results of IPCC 2021 GWP100 (including CO2 uptake) method using high voltage electricity.

**Figure 14:**
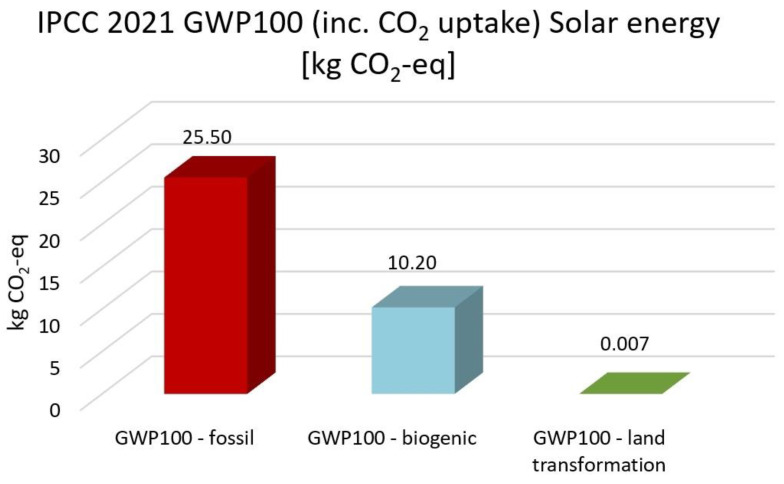
Results of IPCC 2021 GWP100 (including CO_2_ uptake) method using solar energy.

**Figure 15:**
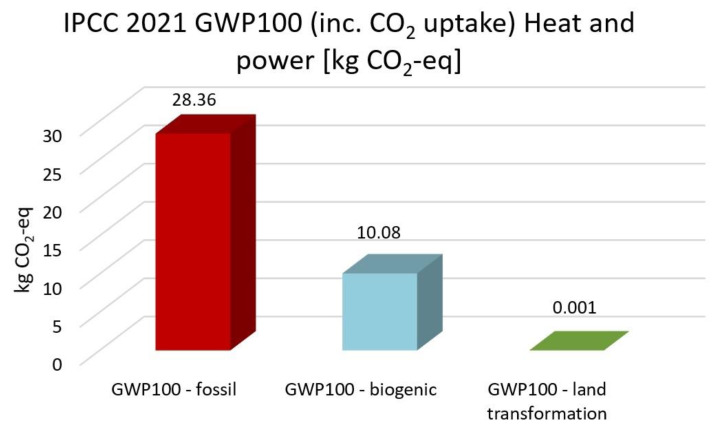
Results of IPCC 2021 GWP100 (including CO_2_ uptake) method using heat and power energy.

**Figure 16:**
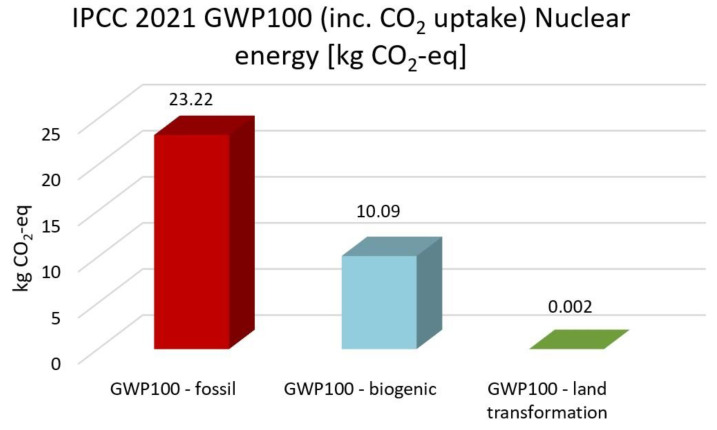
Results of IPCC 2021 GWP100 (including CO2 uptake) method using nuclear energy.

**Figure 17:**
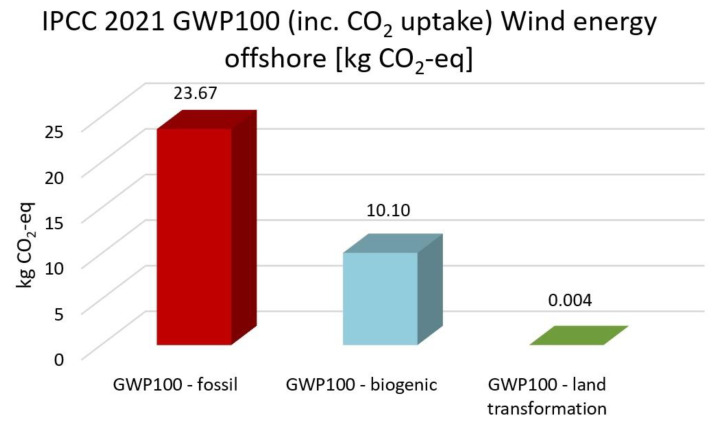
Results of IPCC 2021 GWP100 (including CO_2_ uptake) method using wind offshore energy.

Wind energy onshore had the same results as wind energy offshore. Therefore, it is not depicted. As indicated in Figure 12, Figure 13, Figure 14, Figure 15 and Figure 16, fossil fuels will cause the most significant environmental load with each energy resource, which is consistent with previous findings.

As the results of LCA have variability and uncertainty, our analysis uses various methods to represent the results more deeply. DALY—disability-adjusted life year—is a measure of overall disease burden, expressed as the cumulative number of years lost due to ill health, disability or early death. CTUh—comparative toxic unit for humans—expresses the estimated increase in morbidity in the total human population per unit mass of a chemical emitted (cases per kilogram). Climate change trends focus on the amount of carbon dioxide emissions with the unit of kg⸱CO_2_. Acidification is estimated by sulphur dioxide or hydrogen ion; accumulated exceedance is expressed in mol H^+^ eq. PDF.m^2^.yr quantifies the fraction of species that disappeared forever from a region of the world. It quantifies the temporary disappearance of species (PDF) over a given surface (m^2^) during a certain time (yr). Finally, species.yr refers to the aggregated local loss of species over time (year) [34,35].

### 3.2. Results of the MCDA Analysis

The importance of our analysis is also confirmed by the LCA study of Capa et al. (2022) [36], according to which energy reduction and the development of an appropriate energy policy are extremely important in the operation of wastewater treatment plants.

**Figure 18:**
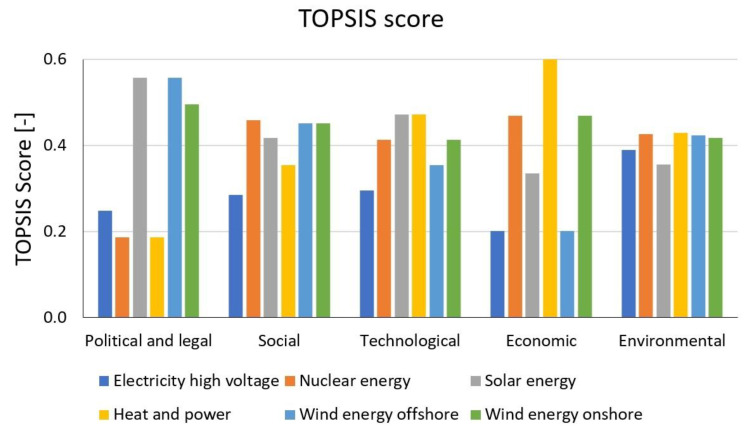
Results of MCDA for PESTLE factors using TOPSIS method, TOPSIS score: the higher the better.

For a comprehensive analysis, it is particularly important to expand the life cycle analysis and evaluate it together with other methods [37]. Multi-criteria decision analysis (MCDA) was conducted to select the most suitable wastewater treatment with different energy process alternatives. A numerical input value for MCDA was taken from the six factors investigated: political, legal, economic, technological, social and environmental. The ranking method was applied to set up weighting between the inspected feedstocks. A TOPSIS score was used to evaluate the alternatives, where a higher score counts better. The results of the MCDA are presented in Figure 18.

The social and environmental factors were derived from the impact assessment results based on the IMPACT+ World endpoint method with a single score, as shown in Table 1. The social analysis shows that nuclear and renewable energies are significantly better than the other two alternatives, while nuclear energy is the best choice. The environmental analysis indicates that heat and power have the lowest negative impact on the environment; however, nuclear and wind energies have attained good scores as well.

In our study, TOPSIS score was used to evaluate the alternatives from the six factors investigated: political, legal, economic, technological, social and environmental. The weight of factors is a subjective input that is not always even and is heavily influenced by the personal perspective of the decision-maker. According to this study, the environmental factor had the greatest weight, followed by social, economic, technological, political and legal factors. Our TOPSIS evaluation showed that wind onshore energy is the best choice. It is green energy, reducing social and environmental impact, and is cost-effective. Wind onshore energy has great potential for development. As far as the actual situation is concerned, European countries are promoting wind onshore energy development. The results of the study and the actual situation are similar. The social analysis showed that nuclear energy is the best choice. Nuclear energy also reduces CO_2_ emissions; it is a clean and reliable energy source. According to Saidi and Omri (2020) the best option to reduce CO_2_ emissions is to use a mix of nuclear and renewable energy. The two sources of energy are complementary [38].

Legal and political review shows that renewable energy provides stable markets for EU countries and contributes to achieving energy policies and goals. The 2030 Climate Target Plan enshrines a minimum 55% reduction in greenhouse gas emissions by 2030 and relies on member states’ national energy and climate plans (NECPs). As part of its pioneering “Delivering the European Green Deal” package, which supported the path outlined in climate law, a series of interconnected proposals was presented across the economy with the aim of increasing the ambition for 2030, among others, through setting new targets for greenhouse gas reduction, renewable energy production and energy efficiency. An EU solar energy strategy was developed by the commission in 2022 to support the innovation and deployment of renewable energy, in which solar and offshore wind energy are most concentrated, followed by onshore wind energy, nuclear and finally high-voltage electricity, heat and power [34,39].

The economic review demonstrates that heat and power are the most economical processes. The economic score of electricity from the remaining energy sources gradually decreases from nuclear, onshore wind, solar PV, offshore wind and high-voltage electricity. The operational costs for membrane contactor-based N and P recovery process are about 2.38 EUR/m^3^, 1.14 EUR/m^3^ for energy costs and 1.24 EUR/m^3^ for chemical costs [1]. Electricity costs may vary by other sources. The average electricity generation cost worldwide in 2021 of concentrating solar power is 0.114 EUR/kWh, solar photovoltaics is 0.048 EUR/kWh, offshore wind is 0.075 EUR/kWh, onshore wind is 0.033 EUR/kWh, nuclear is 0.03 EUR/kWh, all fossil fuels is 0.0076 EUR/kWh [40].

The technological review demonstrates that electricity is the most popular and convenient today. The future trend of technology connecting with solar electricity is evaluated to have potential, meaning solar is given a higher score than other energies.

**Figure 19:**
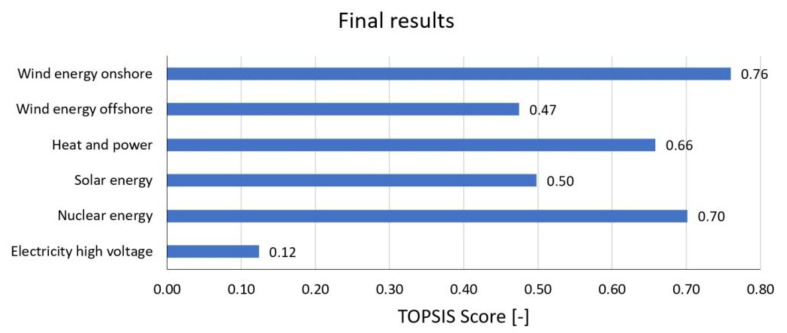
Results of the MCDA.

Figure 19 illustrates the final outcome of the MCDA. For the EU region, the most advantageous alternative is found to be wind onshore, with a TOPSIS score of 0.76, and the next is nuclear, with the value of 0.70.

Kar et al. (2023) [36] performed LCA to investigate the environmental impact of ammonium sulphate fertilizer production by air-stripping ammonia from WWTP sidestreams at varying sidestream nitrogen concentrations. They concluded that air-stripping technology offers an environmentally and economically favorable option for nitrogen recovery and ammonium sulphate production. It is concerned even with varying ammonia concentrations and high sidestream volume. Recovering ammonia can be cost-effective even at low concentrations. However, high ammonia concentration is environmentally beneficial. In their findings, varying flow rates and ammonia concentration can influence the environmental effect of recovering ammonia. According to Kar et al. (2023), renewable energy resources, such as solar and wind, for ammonium sulphate production can significantly reduce fossil-based CO_2_ emissions and environmental impacts. They investigated IPCC global warming for ammonium sulphate produced by Haber Bosch and air-stripping, ReCiPe midpoint indicators for 1 MT ammonium sulphate produced by Haber Bosch and air-stripping processes with various sidestream NH_4_-N concentrations [41]. It can be summarized that their results on environmental impacts are in good accordance with our investigations.

## 4. Conclusions

As a novel side of our research, the gas separation membrane process was investigated with the EF 3.0 method preferred by the European Commission. Furthermore, the environmental analysis was expanded with MCDA-PESTLE analysis.

It can be established from the results that using renewable energy can enormously reduce the environmental impact of nitrogen recovery and fossil-based CO_2_ emission. On the other hand, the storage of renewable energy is problematic. Therefore, it would be realistic to have combined energies that include both renewable and conventional energy resources.

It can be concluded that renewable energy resources cause a significantly lower environmental load compared to traditional energy resources. Examining climate change and global warming show that high-voltage electricity energy sources have the highest value, with 50–59% lower renewable energy resources values. Human toxicity also shows high-voltage electricity energy source as the highest value, with 3–22% lower renewable energy resource values. As for acidification, electricity also has the highest values, with 17–46% renewable energy values. Finally, eutrophication indicates electricity as the highest value, with 35–39% lower renewable energy values. Fossil fuels will also cause the most significant environmental load with each energy resource, consistent with previous findings.

Regarding the results of MCDA for PESTLE factors using the TOPSIS method, the social analysis shows that nuclear and renewable energies are significantly better than the other two alternatives (high-voltage electricity and heat and power). The environmental analysis indicates that heat and power have the lowest negative impact on the environment; however, nuclear and wind energies have attained good scores as well. The environmental analysis implies that heat and power type has the lowest negative impact on the environment. However, nuclear and wind energies have also reached good scores.

According to the final outcome of the MCDA, for the EU region, the most advantageous alternative was found to be wind energy onshore with a TOPSIS score, followed by nuclear energy.

## Figures and Tables

**Table 1:** Summary of impact assessment results using the IMPACT World+ endpoint method.

IMPACT+ World Endpoint	Electricity High Voltage	Nuclear	Solar	Heat and Power	Wind Energy Offshore	Wind Energy Onshore	Unit
Total	138.09	118.91	140.75	122.96	120.00	121.38	EUR2003
Human health	25.35	15.78	17.31	20.46	16.02	16.06	EUR2003
Ecosystem quality	112.73	103.12	123.45	102.50	103.98	105.32	EUR2003
